# Weakly Supervised Learning of 3D Deep Network for Neuron Reconstruction

**DOI:** 10.3389/fnana.2020.00038

**Published:** 2020-07-28

**Authors:** Qing Huang, Yijun Chen, Shijie Liu, Cheng Xu, Tingting Cao, Yongchao Xu, Xiaojun Wang, Gong Rao, Anan Li, Shaoqun Zeng, Tingwei Quan

**Affiliations:** ^1^Wuhan National Laboratory for Optoelectronics-Huazhong, Britton Chance Center for Biomedical Photonics, University of Science and Technology, Wuhan, China; ^2^Ministry of Education (MoE) Key Laboratory for Biomedical Photonics, Collaborative Innovation Center for Biomedical Engineering, School of Engineering Sciences, Huazhong University of Science and Technology, Wuhan, China; ^3^School of Mathematics and Physics, China University of Geosciences, Wuhan, China; ^4^School of Electronics Information and Communications, Huazhong University of Science and Technology, Wuhan, China

**Keywords:** neuron reconstruction, weakly supervised deep learning, precise, generalization, automatic

## Abstract

Digital reconstruction or tracing of 3D tree-like neuronal structures from optical microscopy images is essential for understanding the functionality of neurons and reveal the connectivity of neuronal networks. Despite the existence of numerous tracing methods, reconstructing a neuron from highly noisy images remains challenging, particularly for neurites with low and inhomogeneous intensities. Conducting deep convolutional neural network (CNN)-based segmentation prior to neuron tracing facilitates an approach to solving this problem via separation of weak neurites from a noisy background. However, large manual annotations are needed in deep learning-based methods, which is labor-intensive and limits the algorithm’s generalization for different datasets. In this study, we present a weakly supervised learning method of a deep CNN for neuron reconstruction without manual annotations. Specifically, we apply a 3D residual CNN as the architecture for discriminative neuronal feature extraction. We construct the initial pseudo-labels (without manual segmentation) of the neuronal images on the basis of an existing automatic tracing method. A weakly supervised learning framework is proposed via iterative training of the CNN model for improved prediction and refining of the pseudo-labels to update training samples. The pseudo-label was iteratively modified via mining and addition of weak neurites from the CNN predicted probability map on the basis of their tubularity and continuity. The proposed method was evaluated on several challenging images from the public BigNeuron and Diadem datasets, to fMOST datasets. Owing to the adaption of 3D deep CNNs and weakly supervised learning, the presented method demonstrates effective detection of weak neurites from noisy images and achieves results similar to those of the CNN model with manual annotations. The tracing performance was significantly improved by the proposed method on both small and large datasets (>100 GB). Moreover, the proposed method proved to be superior to several novel tracing methods on original images. The results obtained on various large-scale datasets demonstrated the generalization and high precision achieved by the proposed method for neuron reconstruction.

## Introduction

Neuronal morphology reflects the organization and function of the brain. Digital reconstruction or tracing of 3D tree-like neuronal structures from optical microscopy images is essential for the morphological characterization and analysis of neurons, synaptic integration, phenotype identification, neural circuit building, and network mapping, all of which reveal the role of neurons in brain activities (Donohue and Ascoli, 2011; Parekh and Ascoli, 2013; Peng et al., 2015; Abdellah et al., 2018). Currently, most neurons are reconstructed by hand, which is a laborious, time-consuming, and non-reproducible task (Brown et al., 2011; Wang et al., 2019). Therefore, automatic and accurate neuron reconstruction or tracing methods are in high demand in computational neuroscience.

With advances in optical imaging and molecular labeling techniques, neuronal images were achieved at submicron resolution and at the large scale of a mammalian brain (Li et al., 2010; Jefferis and Livet, 2012; Silvestri et al., 2012; Gong et al., 2013; Xiong et al., 2014; Economo et al., 2016). These advances have fueled the generation of various optical images for different applications and posed new challenges in neuron reconstruction. One such challenge arises from significant variations in the image quality and attributes across different datasets due to several factors, including differences in the imaging system, labeling methods, animal species, neuron types, and individual users (Brown et al., 2011; Chen et al., 2015; Peng et al., 2015). For example, the intensity range (0–255 vs. 0–4095), image size (megabytes to gigabytes vs. terabytes), neuronal structures (single short neurons vs. brain-size long-range projection neurons with complex dendrites and axons) are entirely different for the mouse neuronal images from the BigNeuron datasets (Peng et al., 2015) and the fMOST (fluorescence micro-optical sectioning tomography) datasets (Gong et al., 2013). Another challenge lies in the fact that microscopy images generally have high background noise, exhibiting weak and discontinued neurites (Mukherjee et al., 2015; Li R. et al., 2017; Li S. et al., 2017). It is difficult to discern neurites with low and uneven intensities from the noisy background, particularly for large-scale neuron images with large image blocks and low signal-to-noise ratio (SNR) (Li et al., 2019b; Wang et al., 2019). The variability among different datasets and low SNR of optical images all add to obstacles for the generalization and accuracy of neuron reconstruction algorithms.

Numerous semiautomatic or automatic methods have been proposed for the tasks of neuron reconstruction or tracing of optical images (Rodriguez et al., 2009; Bas and Erdogmus, 2011; Wang et al., 2011; Zhao et al., 2011; Basu et al., 2013; Mukherjee et al., 2013, 2015; Xiao and Peng, 2013; Dercksen et al., 2014; Chen et al., 2015; Quan et al., 2016; Li S. et al., 2017; Radojević and Meijering, 2017; Skibbe et al., 2019). A diversity of computational concepts and various global and local image characteristics have been employed in these algorithms to achieve neuron tracing. These include, but are not limited to, region growing (Rodriguez et al., 2009), open curve snake (Wang et al., 2011), tubular model (Zhao et al., 2011; Feng et al., 2015), all-path pruning (Xiao and Peng, 2013), principal curves (Bas and Erdogmus, 2011; Quan et al., 2016), graph-theoretical approach (De et al., 2016), tubularity flow field (Mukherjee et al., 2015), probability hypothesis density filtering (Radojević and Meijering, 2017), and self-learning-based support vector machine (SVM) (Chen et al., 2015; Li S. et al., 2017). These algorithms generally exhibit good performance on optical neuronal images with clear structures. However, many methods are designed based on specific datasets or for particular problems, and their performances on various types of datasets may decline, whereby complicated parameter adjustments would be required. Furthermore, most algorithms perform poorly with regard to tracing neurites from images with low SNR and tend to either over-reconstruct background noise or under-trace weak neurites (Xiao and Peng, 2013; Chen et al., 2015; Mukherjee et al., 2015).

Recently, deep convolutional neural networks (CNN) have achieved impressive performances in both nature and medical image segmentation (Long et al., 2015; Çiçek et al., 2016; Chen et al., 2018; Falk et al., 2019), owing to their ability to capture richer and more discriminative features than traditional methods. The deep learning toolbox, DeepNeuron (Zhou et al., 2018), was designed for neuron tracing with manually reconstructed neurons as training samples. The algorithm was based on a 2D CNN, and its performance in the case of weak neurites was poor (Zhou et al., 2018). A 3D CNN designed for neuron segmentation and tracing exhibited improved performance on images with high noise (Li R. et al., 2017; Li and Shen, 2019). However, these algorithms demanded a large amount of labor-intensive, time-consuming, and expensive manual annotations for neuron segmentation (Magliaro et al., 2019). Users are required to annotate an adequate number of new samples for neuron datasets from different organizations to achieve robust estimation. The need for manual annotation has significantly limited the generalization of deep learning-based methods for various optical neuron images.

In this study, we propose a weakly supervised deep learning method for automatic neuron reconstruction. A 3D deep residual CNN was employed for accurate neuron detection from neuronal images with low SNR. A weakly supervised deep learning framework was developed to improve the generalization of the CNN for various neuronal datasets without manual annotation. The framework was built via iterative expansion of undetected weak neurites and refining of the training samples for retraining, on the basis of neurite properties of tubularity and continuity. The comparison of results on challenging datasets demonstrates that the proposed method significantly improves the tracing performance of weak neurites from images with high noise and outperforms several novel tracing algorithms on original images. Performances on various large datasets demonstrate the high precision and generalization of the proposed method in neuron detection, which can be used to significantly improve neuron tracing for various large datasets without laborious manual annotation.

## Materials and Methods

**FIGURE 1:**
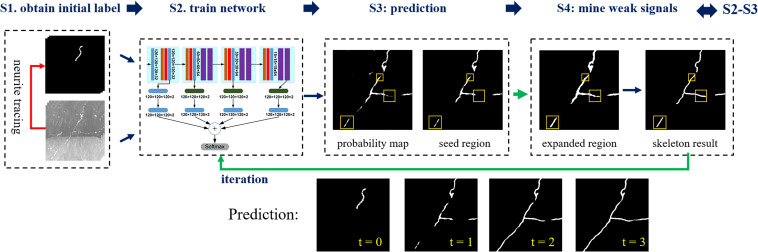
Flowchart of the proposed weakly supervised deep learning method for neurite segmentation. Four steps, S1–S4. S1. Automatic segmenting neurites from optical images using an existing neuron tracing method and tubular neurite shape. S2. Training of a 3D segmentation network using input images and their pseudo-labels (automatic segmentations) as training sets. S3. Predicting the probability map of the foreground via CNN and using the segmentation as seed region. Yellow boxes indicate neurites that not detected by CNN with pseudo labels. S4. Refining pseudo labels of training samples (labeled by yellow boxes): expanding the seed region to include more weak neurites using region growing and obtaining skeleton results to remove background. Iterating steps 2–4 to update pseudo labels of training samples for foreground segmentation improvement (three iterations).

We propose a weakly supervised learning framework of 3D deep CNNs for automatic and accurate neuron tracing without hand-designed features or manual annotations. The flowchart of the proposed framework is illustrated in Figure 1. The method includes four steps: (1) automatic segmentation of neurites from optical images based on an existing automatic tracing method and tubular neurite shape, (2) training of a 3D segmentation network using input images and their pseudo-labels (automatic segmentations) as training sets, (3) prediction of the probability map of the foreground using CNN, and (4) refinement of pseudo-labels of training samples by mining weak neurites from the probability map using region growing and skeleton strategies. Steps 2–4 are iterated to update the pseudo-labels and optimize the segmentation network.

### Initial Training Labels

Initial neurite segmentations are automatically obtained via an existing tracing method and identification of neurite shape characteristics. These are then regarded as the initial training labels (i.e., pseudo-labels). First, neurite skeletons are traced via an existing automatic tracing method. Here, we apply our previously established method ST-LVF for tracing (Li et al., 2019b), which is integrated into the GTree software^1^. In ST-LVF, a local threshold is applied to separate neurite signals from the background, and SVM is employed to identify weak signals for which the local threshold fails (Li et al., 2019b). During tracing, default parameters were chosen and the threshold parameter was set to 1–2 to keep weak signals in the training images. Isolated short neurites or short branches were removed to prevent background noise interference. Subsequently, the 3D skeletons were resampled to maintain the adjacent skeleton points that are connected in their 26-voxel neighborhood. Considering the tubular shape of neurites and their typical radius (2–4 pixels), 3D cylinder shapes with skeletons at their centerline and a radius of two pixels are finally generated as initial neurite segmentations.

### Network Architecture and Loss Function

**FIGURE 2:**
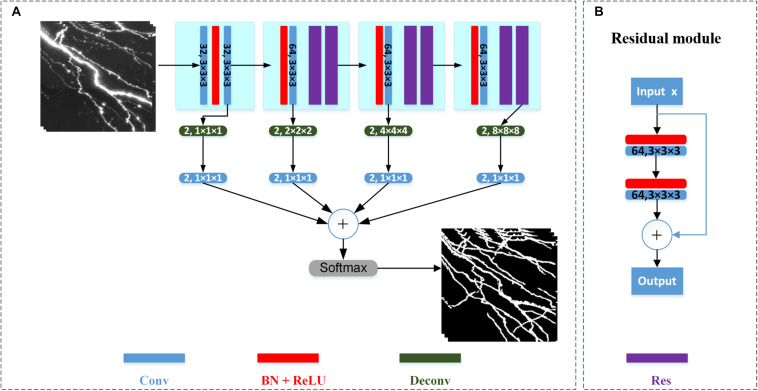
Architecture of VoxResNet for neurite segmentation. **(A)** Detailed network architecture. Sizes of the convolutional (Conv) and deconvolutional (Deconv) layers are shown on the respective boxes. Batch normalization (BN) and rectified linear unit (ReLU) activation function is applied. Res refers to residual module. **(B)** Structure of residual module.

We employ the 3D deep voxelwise residual network (VoxResNet) for neurite segmentation (Chen et al., 2018). The network is chosen owing to its following attributes: (1) It employs the concept of deep residual learning in image recognition to facilitate the training process and improve the accuracy in deep layers (He et al., 2016). (2) It demonstrates robustness and precision in volumetric image segmentation at different scales, as it seamlessly integrates multi-resolution image appearances and context features at low and high levels with deep supervision (Chen et al., 2018). The architecture of the network is illustrated in Figure 2. The network contains 21 convolutional layers (including six stacked deep residual modules) and four deconvolutional layers. The stacked convolutional layers with a kernel size of 3 × 3 × 3 are used to extract features from different receptive field sizes. The number of channels in the first two layers is 32 with stride 1, and the number of channels of other layers is 64 with stride 2. The residual module is shown in Figure 2B. To prevent the optimization degradation problem, the residual module propagates information directly from the forward block to the backward block by applying identity mappings via a shortcut connection and element-wise addition. Because the optimal function is closer to an identity mapping than to a zero mapping, it is considered easier for a solver to drive the weights of multiple nonlinear layers toward zero to approach identity mapping (He et al., 2016). The deconvolutional layers are employed to map the extracted features to pixels using the same functions as convolutional layers, such as filtering and pooling; however, they do so in reverse (Zeiler and Fergus, 2014). The deconvolutional layers comprise two channels (i.e., foreground and background). The deconvolutional layers’ output is processed by convolutional layers of kernel size 1 × 1 × 1 for auxiliary classification, and the classified results are combined for the final output. A softmax layer is finally used to normalize the output in the range [0, 1]. In the network, a rectified linear unit is used as the activation function. Batch normalization, which involves normalization for each training mini-batch to achieve regularization, is applied to reduce the internal covariate shift and improve the prediction.

Three-dimensional patches of 120 × 120 × 120 or 64 × 64 × 64 volumes were randomly chosen as training samples from the training images considering the input image size, computational cost, and segmentation accuracy (Huang et al., 2018). Patches containing few voxels of positive pseudo-labels (<α_1_*V*, where V denotes the total number of voxels of a patch, and α_1_ is a ratio set to 0.001) were removed to eliminate blank patches. The patches were normalized to zero mean and unit variance, and random rotation, flip, contrast and brightness adjustment, and Gaussian blur were performed for data augmentation to enhance network robustness. Stochastic gradient descent optimization was applied. The initial learning rate is 0.01 and decreased by half every four epochs. In the training, the batch size, momentum, and weight decay are set to 3, 0.9, and 0.0005, respectively. The maximum number of training epochs is set to 100.

Because neurites only occupy a small fraction of the patches, the foreground (i.e., segmented neurites) and background classes are usually unbalanced, which could lead to a prediction bias. Weighted cross-entropy loss and dice loss functions mitigate the influence of class imbalance (Huang et al., 2018; Falk et al., 2019). We adopt a hybrid loss function combining the two loss functions to prevent class imbalance and preserve region continuity, which is calculated as follows:

(1) $$\begin{array}{c} l\,o\,s\,s_{\text{hybird}}=\sigma \,l\,o\,s\,s_{\text{cross}\;\text{entropy}}+l\,o\,s\,s_{\text{dice}} \end{array}$$

(2) $$\begin{array}{c} l\,o\,s\,s_{\text{cross}\;\text{entropy}}=\sum_{i=1}^{m}-\alpha \cdot g_{i}\,\log\left(p_{i}\right),\;\alpha =\frac{\sum_{i=1}^{m}g_{i}}{m} \end{array}$$

(3) $$\begin{array}{c} l\,o\,s\,s_{\text{dice}}=1-2\,\frac{\sum_{i=1}^{m}p_{i}\,g_{i}+\varepsilon }{\sum_{i=1}^{m}p_{i}+\sum_{i=1}^{m}g_{i}+\varepsilon } \end{array}$$

where *p*_*i*_ is the predicted probability of pixel *i*; *g*_*i*_ is the corresponding pseudo-label for the foreground or background with a value of 0 or 1, respectively; α is the ratio of the foreground pseudo-label voxel numbers to the image volume voxel numbers *m*; and ε is a smoothing parameter set to 1; σ is used to balance the ratio of the loss functions, and it is set to 0.5.

### Mining Weak Neurites for Pseudo-Label Refinement

**FIGURE 3:**
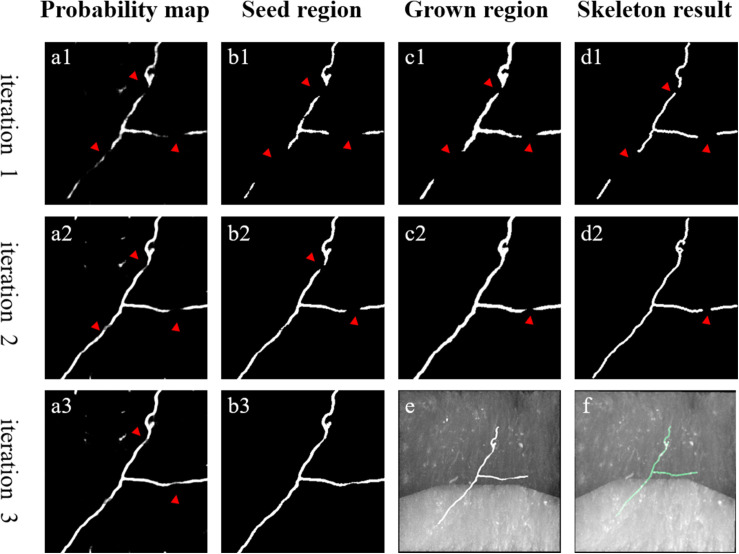
An example of weak neurite mining process for pseudo-label refinement. **(a1–a3)** Estimated foreground probability map by CNN. **(b1–b3)** Seed region of foreground via maximum probability classification and image denoising. **(c1,c2)** Grow the seed region to include nearby weak neurites. **(d1,d2)** Skeleton of the grown region to refine pseudo labels. **(e,f)** Overlapped results of predicted neurites by CNN, manual annotation and original images, respectively. Red arrows point to the changed areas.

In initial training labels obtained by automatic tracing, some weak and inhomogeneous neurites are not detected, which decreases network prediction accuracy for neuronal images with low SNR. Thus, the initial training set must be refined for better segmentation. We first train the network with the initial training set. Then, we iteratively mine more undetected weak neurites from the CNN-predicted probability map based on the neurite tubular and continuous characteristics, employing region growing and skeleton methods. Figure 3 depicts the mining process for pseudo-label refinement.

The detailed mining steps are described as follows.

1. Generation of the seed region of neurites using the network with the maximum probability classification. Removal of small objects (volume size < 200 voxels) to prevent noise interference.
2. Expansion of the seed region to include weaker neurites using region growing based on the neurite continuity. *O*_*reg*_ represents the seed region. Then, for each voxel in *O*_*reg*_, its neighborhood is searched according to (4):

(4) $$\begin{array}{c} G_{\text{reg}}=\left\{v\in N\,\left(v^{\text{o}}\right)|s\,\left(v\right)>t\,h\,r\,e,v^{\text{o}}\in O_{\text{reg}}\right\} \end{array}$$

where *v*^*o*^ and *v* represent voxels; *N*(*v*^*o*^) is the 8-voxel neighborhood of *v*^*o*^; *s*(*v*) is the probability map value of *v*; *thre* is an adaptive threshold for region growing; and *G*_reg_ is the grown region of *G*_*reg*_.

After obtaining *G*_reg_, *O*_reg_ is replaced with *G*_*reg*_ and *G*_*reg*_ is updated according to (4). This procedure is repeated until *G*_*reg*_ converges. During region growing, *thre* remains fixed as the average value of the neighborhood voxels in the seed region. The neighborhood is defined as:

(5) $$\begin{array}{c} N_{\text{reg}}=\left\{v\in N^{*}\,\left(v^{\text{o}}\right)|v\notin O_{\text{reg}},v^{\text{o}}\in O_{\text{reg}}\right\} \end{array}$$

where *N*^∗^(*v*^o^) is the 124-voxel (124 is 5 to the power 3 minus 1) neighborhood of *v*^o^. In comparison to the seed region, the grown region contains more voxels from the neurite region.

3. Extraction of the closely connected skeleton of the grown region via a thinning method (Lee et al., 1994). Removal of short branches to prevent spurious end nodes caused by irregularities along the surface of the grown region (Rodriguez et al., 2009).
4. Update of the neurite region according to the extracted skeleton. The 3D cylinder objects with the skeletons as the centerline and a radius of two are obtained as updated neurite segmentations. Thus, new pseudo-labels are generated.

**FIGURE 4:**
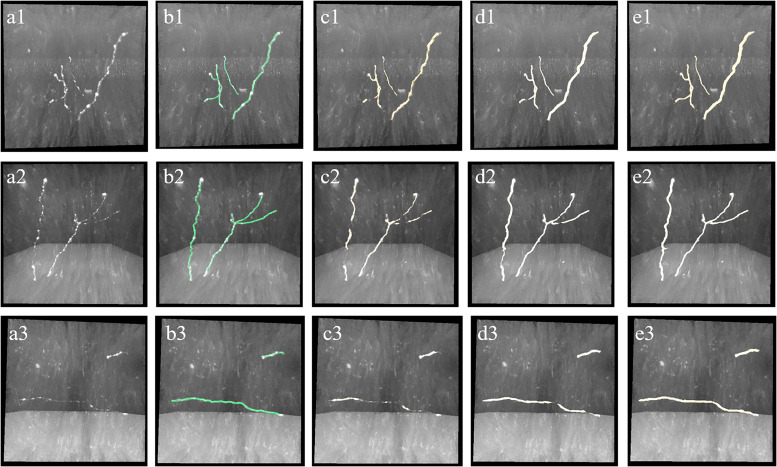
Iterative process of the proposed method for weak neurites mining. Column **(a)** shows typical neuronal images with low and uneven foreground. Column **(b)** shows manual tracing results. Columns **(c–e)** show overlapped results of predicted neurites by CNN and original images of the first, second, and third iterations, respectively.

The proposed weak neurite mining process allows the update of training labels. The updated training set is used to retrain the CNN for better prediction of weak neurites, and more undetected weak neurites are mined from the newly predicted probability map. This procedure is repeated until the pseudo-labels of the training set converge or the iteration number becomes larger than a defined maximum iteration (between 3 and 5). This iterative process is illustrated in Figure 4 (the first iteration indicates the performance of the first trained CNN with initial training labels that are generated by the automatic tracing method).

### Post-processing

In this study, we enhance the original optical images using the CNN-predicted probability map to suppress image noise and strengthen weak neurites. The predicted probability map has the same size as the input image stack with values between [0, 1]. The enhanced procedure is similar to the method in Li R. et al. (2017) and calculated as follows:

(6) $$\begin{array}{c} F\,\left(x\right)=\delta \cdot I\,\left(x\right)+\left(1-\delta \right)\cdot I_{\text{M}}\cdot P\,\left(x\right) \end{array}$$

Here, *I*(*x*), *P*(*x*), and *F*(*x*) represent the value of the original image, predicted neurite probability map, and enhanced image at voxel *x*, respectively; *I*_M_ is the maximum intensity of the image and δ is a weight parameter, and it is set between 0.6 and 0.8.

After neuronal image enhancement, the previously employed ST-LVF method is applied on the enhanced image for neurite tracing (Li et al., 2019b) with default parameters. The above procedure yields the tracing performance from the enhanced image as the result of the proposed method and compares it to other reconstruction methods performed on original images.

### Evaluation

For an objective and systematic evaluation of neuron reconstruction or tracing algorithms, typical criterions of precision and recall (Quan et al., 2016; Li S. et al., 2017; Li et al., 2019b), as well as neuron distance criterions ESA12 (entire-structure-average distance from neurons 1 to 2), ESA21 (entire-structure-average distance from neurons 2 to 1), ESA (average of the bidirectional entire-structure-average distance), DSA (different-structure average), and PDS (percentage of different structures) (Yang et al., 2018), are employed for quantitative assessment. The manual tracing result of the skeletons is considered as the gold standard. Precision and recall are defined as the ratio of true-positive skeleton points to the total number of skeleton points obtained from tracing algorithms and manual reconstruction, respectively. For any skeleton point obtained from the tracing algorithm, if the distance from the nearest point obtained from manual reconstruction is below n pixels (n is set to six considering the neurite radius), the skeleton point is regarded as a true positive (Quan et al., 2016; Li S. et al., 2017). High-precision and recall values correspond to a good segmentation result. For the five-neuron distances, neuron 1 is set to the gold standard and neuron 2 is set to the tracing result by algorithm. The distances can be calculated from a neuron distance plugin in Vaa3D with default distance threshold 2 (Yang et al., 2018). In contrast to precision and recall, lower neuron distances correspond to a better segmentation result. Before evaluation, the skeletons obtained by tracing algorithms and manual reconstruction are equally resampled to maintain the distance between any two neighboring skeleton points as one pixel.

### Experimental Setup

We evaluated the proposed method and several best available tracing algorithms (Rodriguez et al., 2009; Xiao and Peng, 2013; Li et al., 2019a) on various 3D optical neuronal datasets, including fMOST datasets for brain-scale long-projection neuron images at terabyte scale (Gong et al., 2013), and the public BigNeuron (Peng et al., 2015) and Diadem Challenge datasets (Brown et al., 2011).

For the fMOST datasets, we selected image stacks from brain-scale mouse neuronal images with a voxel size of 0.2 × 0.2 × 1 μm for training and testing. The training set included 35 image stacks of 300 × 300 × 300 volume from different brain regions. The testing set included 15 challenging image stacks of volume 300 × 300 × 300, five image stacks of volume 1000 × 1000 × 300, and a large image (approximately 140 Gigabytes) of volume 9620 × 3780 × 2100. BigNeuron and Diadem datasets comprised neuronal image stacks from different organizations of various types, scales, and sizes (Brown et al., 2011; Peng et al., 2015). We validate the generalization and accuracy of the proposed method on the public datasets using transfer learning. In our weakly supervised training process, manual tracing results of the public datasets were not used. A larger neuronal image from the datasets of volume 2111 × 3403 × 291 and voxel size of 0.18 × 0.18 × 0.5 μm was used for fine-tuning, and six image stacks with voxel sizes of 0.31–1, 0.31–1, and 0.54–3.4 μm were used for evaluation.

The proposed method was implemented on C++ and Python 3.6 using the PyTorch library. The segmentation network was trained and evaluated on a computer with Intel i7-6850K CPU (64 GB RAM) and two NVIDIA 1080Ti GPUs.

## Results

**FIGURE 5:**
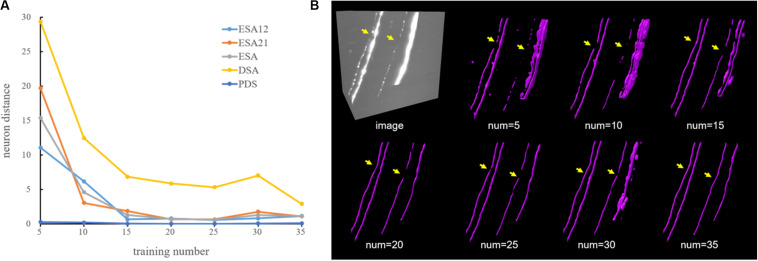
Comparative performance of a deep learning method using different quantities of manual annotation on neuronal images. **(A)** Evaluation results using five-neuron distance. **(B)** An example of CNN-predicted segmentations using different numbers of training samples. Yellow arrows indicate differences across different experiments.

A general deep learning method (GDL) is normally regarded as the “upper bound” of a weakly supervised deep learning method. A GDL is applied on the same network and training samples as the proposed method, while neurite labels were manually annotated and not generated automatically like in the proposed method. Generally, a GDL demands laborious and expensive manual annotation for robust estimation, which limits its use for various datasets. Here, experiments using different quantities of manual annotation had been performed to justify the work of the proposed method and explain why it is urgently needed in deep learning method. In the experiments, 35 neuronal images from different brain regions were carefully selected as training dataset, which included neuronal images with different neurite appearances (neurites with high, low, or inhomogeneous intensities) and structures (straight or twisted neurites with varied radius). We randomly selected 5, 10, 15, 20, 25, 30, and 35 images from the training dataset as training samples. The average neuron distance evaluation results of images in Figure 7 were showed in Figure 5A. The distances were large when only using few training samples since the network trended to overfit with a small number of samples. As the number of training samples grew, the neuron distances stepped down and the estimations became closer to the ground truth (for training number 35, all the distances were lower than 3). As shown in Figure 5B, the network was hard to estimate neurites with weak and inhomogeneous intensities or extreme high intensities accurately using only a few samples. The performances became more accurate and robust with more quantities of manual annotations. These results suggest that large numbers of training samples are needed for robust neurite segmentation, especially for neurites from large-scale images with varied intensities and structures.

**FIGURE 6:**
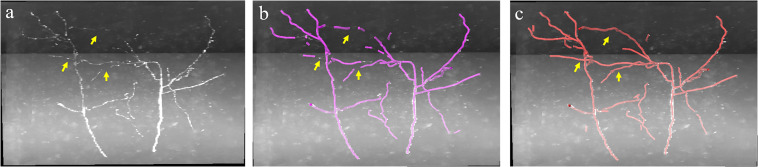
Comparative performance of the proposed weakly supervised method without/with weak neurite mining process for images with low SNR. **(a)** Original image; **(b,c)** represent tracing performance without and with the mining process. Yellow arrows indicate weak neurites.

In the proposed weakly supervised deep leaning method, the iterative mining process for training label refinement is key to neurite segmentation from images with low SNR. Figure 4 shows the comparative neurite detection results using the proposed method with no iterative mining process (third column, the first performance of the trained CNN with initial training labels) and with the iterative mining process (fourth and fifth columns). Without the iterative mining process, the trained network had difficulties in detecting neurites with low and uneven intensities from a noisy background, which led to incomplete or failed tracing of these neurites. With the proposed iterative mining process, these difficult-to-identify neurites can be detected by the final trained network, whose performance is compared to the first trained CNN. We further evaluated the role of the weak neurite mining process using the same iterative procedure with and without the mining process on five fMOST image stacks of 1000 × 1000 × 300 volume. Without the mining process, some neurites with weak and uneven intensities remained undetected, and the tracing results were discontinuous, requiring extensive manual proofreading (Figure 6b). In contrast, with the mining process, the proposed method detected almost all neurites (Figure 6c). The entire skeleton length of the neurites in the five image stacks with and without the mining process was likewise calculated. The average skeleton lengths increased by 212.0 μm when using the mining process, representing 13.2% of the detected neurite length. Hence, the mining process is essential to the proposed method of accurate neurite detection in images with low SNR.

**FIGURE 7:**
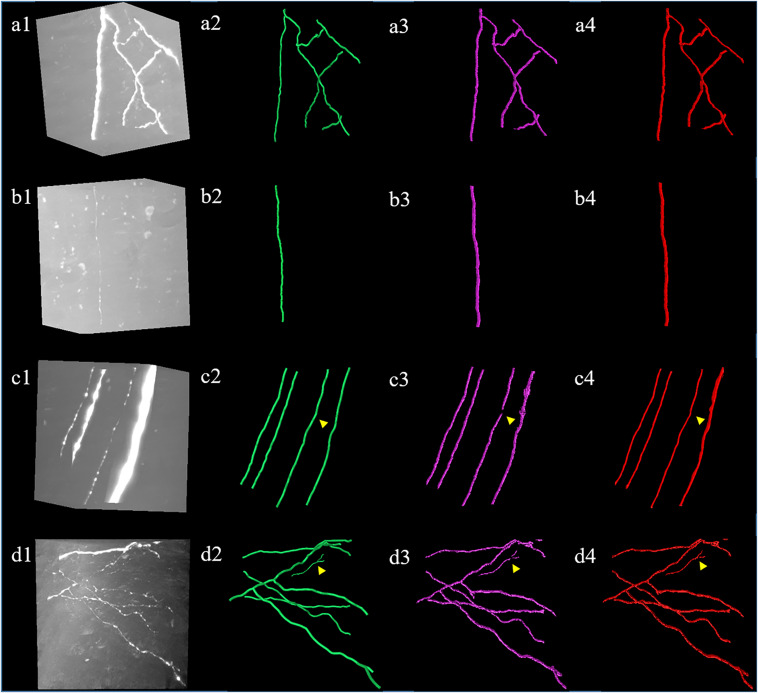
Comparative results of the proposed weakly supervised **(a3–d3)** and a general deep learning method with same network **(a4–d4)** and manual annotations **(a2–d2)**. **(a1–d1)** show neuronal images with different intensity distributions and noise level. Yellow arrows indicate small detection differences between two methods.

We evaluated the effectiveness and accuracy of the proposed weakly supervised deep learning method for neurite detection and tracing by two approaches: (1) comparing the detection performance of the proposed and a GDL, (2) comparing the tracing performance of the proposed method with several best available tracing methods (Rodriguez et al., 2009; Xiao and Peng, 2013; Li et al., 2019b). Figure 7 shows the performances of the proposed and GDL method on neuronal images with various intensity distributions and high noise. Both the proposed and GDL methods demonstrated accurate detection of neurites from neuronal images with different appearances, and their detection difference was very small (as pointed out by the yellow arrows in Figure 7, only small, very thin, and weak neurites were not detected by the proposed method). The quantitative detection results (neurite skeletons) of the two methods were evaluated on 10 neuronal image stacks of 300 × 300 × 300 volume. The recall and precision were 98.4 and 98.5% for the proposed method and 99.6 and 99.8% for the GDL method, respectively. The mean value and standard deviation of ESA12, ESA21, ESA, DSA, and PDS distances in images shown in Figure 7 are listed in Table 1. Neuron distances of both the weakly supervised and manually annotated deep learning methods were small (ESA ≤ 1.2 and PDS ≤ 0.1), and their differences were smaller than 0.06. These results suggest that the proposed method achieves a comparable detection performance to the GDL method and manual detection, without the need for human annotation.

**TABLE 1:** The mean (standard deviation) of ESA12, ESA21, ESA, DSA, and PDS distances between gold standard reconstructions, weakly supervised (our), and manual-annotated deep learning method on images in Figure 7.

Metrics	ESA12	ESA21	ESA	DSA	PDS
**Methods**					
Weakly supervised	1.20 ± 0.26	1.17 ± 0.25	1.19 ± 0.25	2.88 ± 0.44	0.09 ± 0.09
Manually annotated	1.17 ± 0.35	1.11 ± 0.25	1.14 ± 0.30	2.92 ± 0.74	0.10 ± 0.09

**FIGURE 8:**
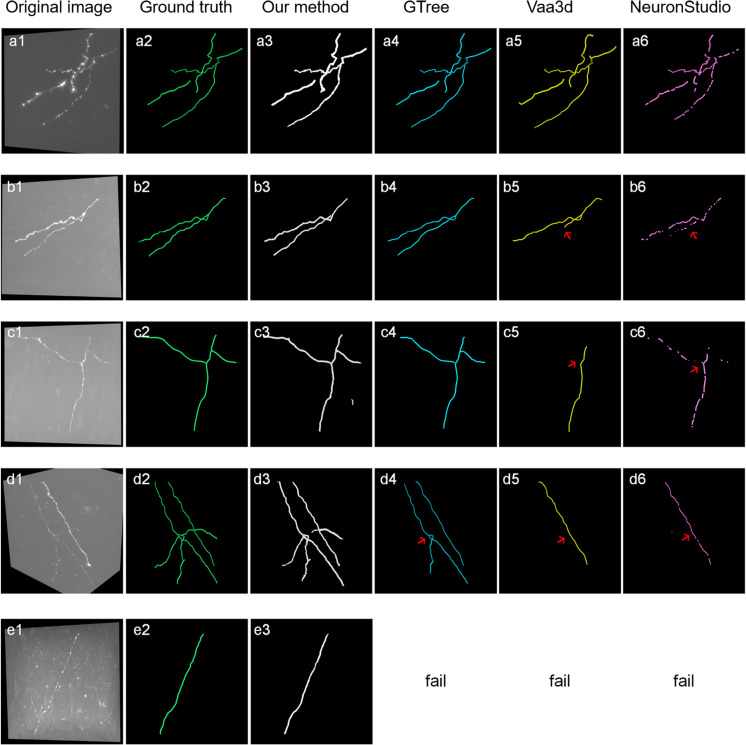
Comparison of the proposed method and several best available tracing methods on neurite tracing. **(a1–e1)** show different original neuronal images. **(a2–e2)** show corresponding manual tracing results. **(a3–e3)**, **(a4–d4)**, **(a5–d5)** and **(a6–d6)** are the tracing results of the proposed method, ST-LVF (GTree software), APP2 (Vaa3d software) and voxel scooping method (NeuronStudio software). Red arrows indicate untraced weak neurites by tracing methods from images with low SNR.

Figure 8 shows the tracing performance of the proposed method and several novel semiautomatic and automatic tracing methods on five neuronal images with weak and inhomogeneous intensity neurites of 300 × 300 × 300 volume. Tracing methods include the voxel scooping method (Rodriguez et al., 2009), the APP2 method (Xiao and Peng, 2013; Peng et al., 2014), and the previously employed ST-LVF method (Hang et al., 2017; Li et al., 2019b). To achieve a fair comparison, several parameters were carefully set to separate neurites from a noisy background and obtain the optimal tracing results. These included the parameter for global threshold estimation, initial seed points of the voxel scooping algorithm, the parameter for global background value estimation of the APP2 algorithm, and the parameter for local background value estimation of the ST-LVF algorithm. Tracing by the proposed method was fully automatized without parameter tuning, as described in section “Post-processing.” As pointed out by the arrows in Figure 8, the voxel scooping method (Rodriguez et al., 2009), which employed the dynamic threshold method for neurite segmentation and voxel scooping for tracing, was unable to detect neurites with weak or sudden changes in intensity, rendering the tracing results incomplete and discontinuous. The APP2 method (Xiao and Peng, 2013), which employed a global threshold for the initial neuron segmentation and all-path pruning for refinement, likewise faced difficulty in tracing some weak and uneven neurites from images with high noise. The ST-LVF method, which employed a threshold-based method for initial neurite detection and a SVM-based method for the detection of weak neurites, detected more neurites than the voxel scooping and APP2 methods, while still failing to trace some weak neurites from the noisy background. The proposed method, employing weakly supervised learning for automatic training of label building and the deep learning method for the acquisition of more representative features, succeeded in the detection of almost all weak neurites and achieved very similar results to the manual detection. The quantitative tracing performance of the corresponding images in Figure 8 is shown in Table 2. The average recall and precision of the proposed method were 0.996 and 0.998, respectively, which were significantly higher than the recall 0.788–0.800 and precision 0.490–0.730 of other tracing methods (Rodriguez et al., 2009; Xiao and Peng, 2013; Li et al., 2019b). The mean value and standard deviation of ESA12, ESA21, ESA, DSA, and PDS distances of images in Figure 8 are listed in Table 3. With the proposed method, tracing could be performed on all five images, while other methods failed to trace neurites from images with low SNR. Our method exhibited the smallest average mean and standard deviation of ESA12, ESA, and DSA distances, with values close to zero. The results demonstrate that the proposed method promotes weak neurite tracing and considerably outperforms several novel tracing algorithms on images with low SNR.

**TABLE 2:** Evaluation of proposed and several best available methods in neurite tracing on five images in Figure 8.

Data	Recall				Precision			
Name	Our	ST-LVF	APP2	Voxel scooping	Our	ST-LVF	APP2	Voxel scooping
a1	**1**	**1**	0.993	0.580	0.996	**1**	0.978	1
b1	0.995	**1**	0.711	0.868	**1**	**1**	0.982	0.998
c1	**1**	0.937	0.461	0.795	**1**	**1**	**1**	**1**
d1	**0.983**	0.747	0.285	0.296	0.993	**1**	0.978	**1**
e1	**1**	0	0	0	**1**	0	0	0
Average	**0.996**	0.737	0.490	0.508	**0.998**	0.800	0.788	0.800

**TABLE 3:** The mean (standard deviation) of ESA12, ESA21, ESA, DSA, and PDS distances between gold standard reconstructions and tracing performances of several best available methods on images in Figure 8.

Metrics	Available number	ESA12	ESA21	ESA	DSA	PDS
**Methods**						
Our	5	**1.30 ± 0.35**	1.26 ± 0.26	**1.28 ± 0.30**	**3.09 ± 0.84**	0.13 ± 0.07
ST-LVF	4	6.46 ± 7.29	**1.19 ± 0.90**	3.83 ± 3.95	8.37 ± 9.35	0.20 ± 0.21
APP2	4	83.56 ± 108.76	4.09 ± 4.43	43.82 ± 56.51	49.47 ± 55.93	0.56 ± 0.30
Voxel scooping	4	24.98 ± 18.67	1.46 ± 2.16	13.22 ± 9.34	29.10 ± 31.91	**0.04 ± 0.28**

**FIGURE 9:**
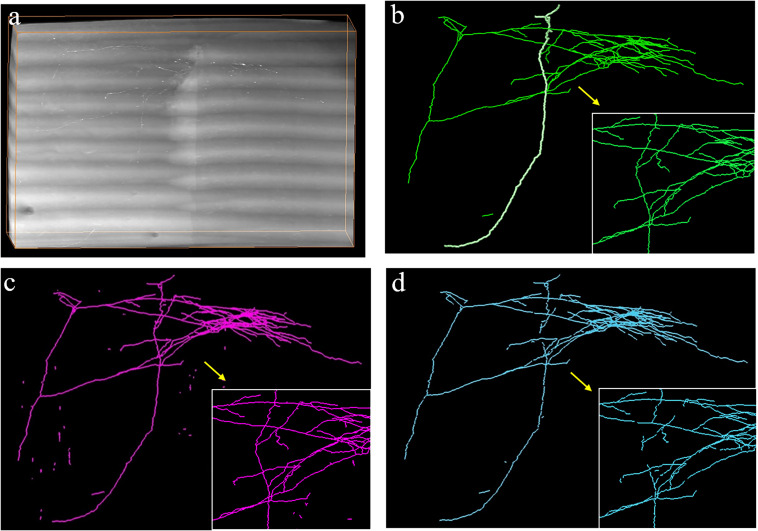
Automatic tracing performance of the proposed method and its basic tracing method ST-LVF on a large-scale dataset. **(a)** Original image with weak neurites and complex neuronal structures. **(b–d)** are performances of manual tracing, the proposed method, and ST-LVF, respectively. Magnified views are shown in the bottom-right corner of **(b–d)** for better visualization.

We also evaluated the generalization of the proposed method for neurite detection and tracing by two approaches: (1) evaluating the performance of the proposed method on a large-scale dataset (approximately 140 gigabytes, 9620 × 3780 × 2100 volume) with various intensity distributions and (2) applying the proposed method to the public BigNeuron and Diadem datasets (Brown et al., 2011; Peng et al., 2015), which have entirely different styles of data compared to the fMOST datasets (Gong et al., 2013). Figure 9 shows the tracing results of the proposed method and its basic tracing method ST-LVF (Li et al., 2019b) on a large-scale dataset that included numerous weak neurites and complex structures. To process this large dataset, we first partitioned the dataset into small sequential image blocks, after which we applied the automatic tracing method ST-LVF on the original image and its corresponding enhanced image using the proposed method. We finally joined the tracing results in the sequence for large-scale neurite tracing. The proposed method achieved similar results to manual tracing on the large dataset. The recall of the proposed method was 0.991, and for the ST-LVF method it was 0.909. We further evaluated the tracing results of a single neurite highlighted in Figure 9b. There were 28 separations along the highlighted neurite with the ST-LVF method, whereas no separations were obtained by the proposed method. In comparison to the basic ST-LVF method, the proposed method accurately detects more weak neurites from a noisy background, thus improving trace completion and significantly reducing laborious manual neurite tracing work for images with low SNR. The high recall achieved by the proposed method on both small and large datasets confirms the generalization of the proposed method for neuronal images with various appearances and volumes. Notably, a few scattered segments were erroneously regarded as detected neurites by the proposed method (Figure 9c). However, they were easily identifiable and removed as they were isolated, short, and far from other neurites.

**FIGURE 10:**
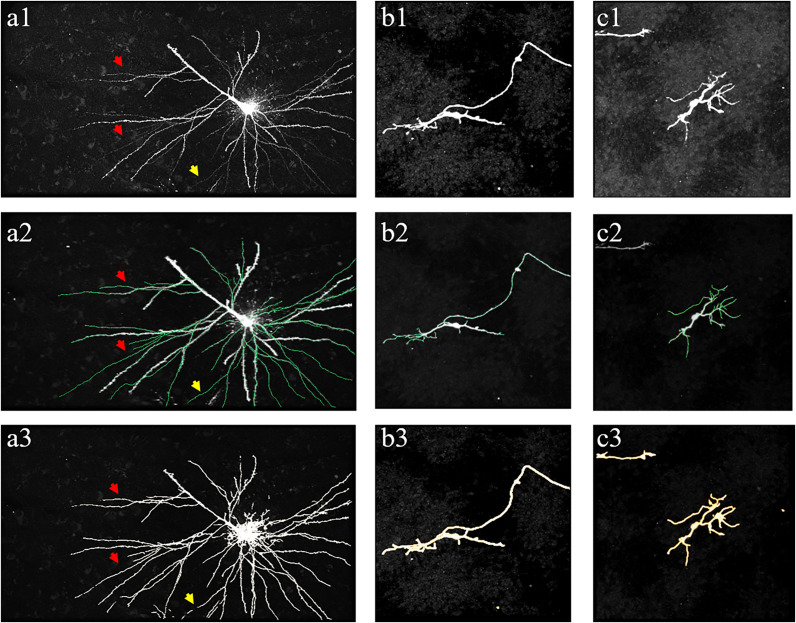
Performance of proposed method on public BigNeuron datasets of human Allen Brain Atlas. **(a1–c1)** show original images. **(a2–c2)** shows manual tracing results (green line). **(a3–c3)** show segmentations of the proposed method. Arrows indicate thin neurites with extreme weak intensity.

**FIGURE 11:**
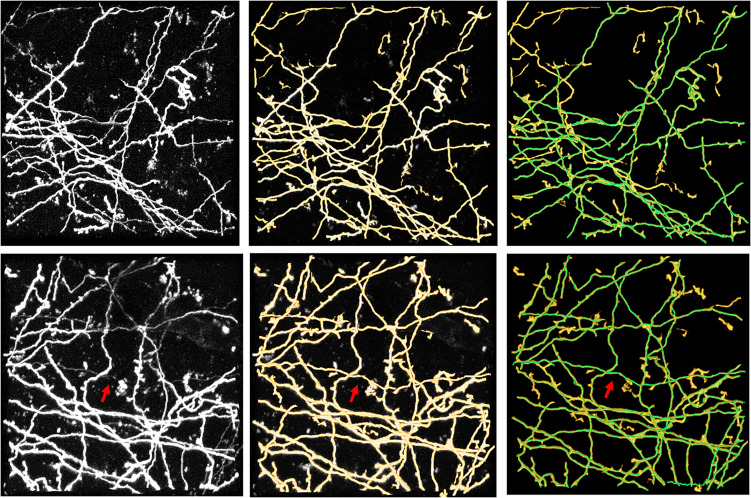
Performance of the proposed method on public Diadem datasets of Neocortical Layer 1 Axon images. The first column shows original images. The second and third columns show segmentations of the proposed method and its combination with manual identification (green line), respectively. Arrows indicate hard-to-identify weak and thin neurites.

Figures 10, 11 show performances of the proposed method on the public BigNeuron and Diadem datasets to demonstrate its generalization on different types of datasets. Because the image format, quality, and attributes vary significantly across public datasets and the previously trained fMOST datasets, we applied transfer learning to reduce the training energy and time spent on new datasets. Unlike traditional transfer learning, which adapts a pre-trained network to new datasets using manually annotated datasets (Falk et al., 2019), the training labels for new datasets were automatically constructed, and the training process was conducted by the proposed method as previously described. Figure 10 shows the segmentations obtained from the proposed method and manual identification on three confocal image stacks from BigNeuron datasets (Peng et al., 2015) with high noise and weak neurites. Figure 11 displays the segmentations of the proposed method on two two-photon laser-scanning image stacks from Diadem datasets (Brown et al., 2011) with densely distributed and uneven intensity neurites on a noisy background. The proposed method detects almost all neurites in the neuronal images, including the hard-to-identify thin neurites with relatively low intensity. These results indicate that the proposed method can automatically learn weak features of new datasets, and it is easily transferred to datasets with varying image formats and qualities.

## Discussion

This paper presents a weakly supervised deep learning method of CNN for automatic neuron reconstruction in various 3D optical images with low SNR. The weakly supervised learning method builds initial training labels using automatic tracing methods and neurite shape characteristics, then iteratively updates training labels by mining more weak neurites to refine the 3D CNN model based on the tubularity and continuity of neurites. In summary, the main contributions of the proposed method are as follows. (1) An accurate, automatic, and general method is proposed for neuron tracing from 3D optical images with low and uneven signal intensities. (2) A weakly supervised learning framework of 3D residual CNNs is presented to address the challenges faced by current methods for precise tracing on different kinds of neuronal datasets. The proposed method demands no special hand-designed features across diverse datasets and no manual annotation for supervised learning algorithms. To the best of our knowledge, this is the first weakly supervised deep learning method to address the problem. (3) The proposed method outperforms several novel tracing methods in weak neurite tracing from highly noisy images and improves the current tracing method. It is effective on both public and fMOST datasets and can perform large-scale and even brain-wide neuron tracing.

Robust and accurate neuron tracing from 3D optical images with low SNR remains a challenge for most methods. Unlike traditional methods, which demand careful hand-designed features and parameter tuning, the deep learning-based algorithm provides an effective and automatic approach for neurite detection from a high-noise background. However, large numbers of training samples are generally needed for accurate and robust estimation when using a deep learning method (Figure 5). The scarcity of manually annotated training samples limits the use of the deep learning-based method for neurite detection across datasets. Herein, a weakly supervised deep learning method is presented to deal with the above issues without manual annotations, which is achieved by three approaches. (1) Initial training labels were built based on an existing automatic tracing method and neurite structure characteristics, which allow the employed 3D CNN to learn discriminative features of the neurites and background. (2) The CNN model is refined by iteratively optimizing the training labels and retraining the model for improved prediction. This process can tolerate some uncertainties during initialization and promote the learning accuracy (as shown in Figures 4, 6). (3) Undetected weak neurites are mined from the probability map of the previously trained CNN model using region growing, skeleton extracting, and neurite structure information. This mining process helps find the neurites that are hard to identify by most tracing algorithms and modifies the training labels to be almost equal to manual annotations, thus improving the tracing accuracy and achieving comparable performance to the network with manual labels (Figures 6, 7). As shown in Figures 8, 9, the proposed method, which assumes the SVM-based ST-LVF tracing method (Li et al., 2019b) as the baseline, enables the detection and tracing of commonly occurring weak neurites and reduces laborious manual correction work of the original tracing method (particularly for large datasets). The comparative performance of the proposed and several novel tracing methods (Rodriguez et al., 2009; Xiao and Peng, 2013; Li et al., 2019b) demonstrated the superiority of the proposed method on neuron reconstruction from images with low SNR. As shown in Figure 9, the proposed method can be applied to large-scale datasets that contain various neuronal structures and weak neurites without parameter tuning. The performance of the proposed method on the Diadem and BigNeuron datasets demonstrates its adaptability to datasets collected from different imaging systems, and no manual annotations are needed for new training samples (Figures 10, 11). Therefore, the proposed method is suitable for numerous types of neuronal images in automatic neurite tracing, and it can be extended to other tracing tasks, such as vessel detection, considering the similar shape characteristics.

Nevertheless, some limitations remain. First, the proposed method is designed for neurite detection and tracing, and the detection of neuronal somas is not as accurate. Specific methods are required to relocate somas from the detection results. Second, although the proposed method is not limited to a specific existing automatic tracing method, the tracing accuracy will affect the number of iterations required for training, and a very low tracing accuracy may undermine the final detection accuracy.

## Conclusion

We propose an accurate and general weakly supervised 3D deep learning-based method for fully automatic neuron tracing without manual annotation. We employed a 3D deep residual CNN for weak neurite detection from a high-noise background and proposed a weakly supervised learning framework to adapt the CNN model to different kinds of optical neuronal datasets without manual labeling, model redesign, or parameter adjustment. The proposed framework exploits the existing automatic tracing method, 3D CNN model, and the tubular and continuous structural characteristics of the neurites to iteratively and automatically refine the training labels of the CNN model. The performances on challenging 3D optical images from different types of datasets demonstrate the accuracy and generalization of the proposed method in neurite detection and tracing. The proposed method outperformed several current algorithms in neurite tracing from images with low SNR and enhanced the current method to achieve more precise and complete neurite tracing. Promising results on a large neuronal image (>100 gigabytes) indicate the potential of the proposed method for long-range projected neuron reconstruction at a large scale.

## Data Availability Statement

The datasets generated for this study are available on request to the corresponding author.

## Author Contributions

SZ and TQ conceived the project. QH and TQ designed the algorithm. QH and YC wrote the manuscript. TQ and YX corrected the manuscript. YC, SL, CX, and TC performed the image analysis. XW, GR, SZ, and AL produced the dataset. All authors contributed to the article and approved the submitted version.

## Conflict of Interest

The authors declare that the research was conducted in the absence of any commercial or financial relationships that could be construed as a potential conflict of interest.

## Footnotes

1 https://github.com/GTreeSoftware/GTree/releases
